# Delivery of costimulatory blockade to lymph nodes promotes transplant acceptance in mice

**DOI:** 10.1172/JCI159672

**Published:** 2022-12-15

**Authors:** Jing Zhao, Sungwook Jung, Xiaofei Li, Lushen Li, Vivek Kasinath, Hengcheng Zhang, Said N. Movahedi, Ahmad Mardini, Gianmarco Sabiu, Yoonha Hwang, Vikas Saxena, Yang Song, Bing Ma, Sophie E. Acton, Pilhan Kim, Joren C. Madsen, Peter T. Sage, Stefan G. Tullius, George C. Tsokos, Jonathan S. Bromberg, Reza Abdi

**Affiliations:** 1 Transplantation Research Center and; 2 Renal Division, Brigham and Women’s Hospital, Harvard Medical School, Boston, Massachusetts, USA.; 3 Department of Surgery and; 4 Center for Vascular and Inflammatory Diseases, University of Maryland School of Medicine, Baltimore, Maryland, USA.; 5 IVIM Technology, Daejeon, South Korea.; 6 Wellman Center for Photomedicine, Massachusetts General Hospital, Boston, Massachusetts, USA.; 7 Institute for Genome Sciences and; 8 Department of Microbiology and Immunology, University of Maryland School of Medicine, Baltimore, Maryland, USA.; 9 Stromal Immunology Group, Laboratory for Molecular Cell Biology, University College London, London, United Kingdom.; 10 Graduate School of Nanoscience and Technology and; 11 Graduate School of Medical Science and Engineering, Korea Advanced Institute of Science and Technology, Daejeon, South Korea.; 12 Center for Transplantation Sciences, Department of Surgery,; 13 Division of Cardiac Surgery, Department of Surgery, and; 14 Division of Transplant Surgery and Transplant Surgery Research Laboratory, Department of Surgery, Brigham and Women’s Hospital, Harvard Medical School, Boston, Massachusetts, USA.; 15 Division of Rheumatology and Clinical Immunology, Beth Israel Deaconess Medical Center, Harvard Medical School, Boston, Massachusetts, USA.

**Keywords:** Transplantation, Organ transplantation

## Abstract

The lymph node (LN) is the primary site of alloimmunity activation and regulation during transplantation. Here, we investigated how fibroblastic reticular cells (FRCs) facilitate the tolerance induced by anti-CD40L in a murine model of heart transplantation. We found that both the absence of LNs and FRC depletion abrogated the effect of anti-CD40L in prolonging murine heart allograft survival. Depletion of FRCs impaired homing of T cells across the high endothelial venules (HEVs) and promoted formation of alloreactive T cells in the LNs in heart-transplanted mice treated with anti-CD40L. Single-cell RNA sequencing of the LNs showed that anti-CD40L promotes a Madcam1^+^ FRC subset. FRCs also promoted the formation of regulatory T cells (Tregs) in vitro. Nanoparticles (NPs) containing anti-CD40L were selectively delivered to the LNs by coating them with MECA-79, which binds to peripheral node addressin (PNAd) glycoproteins expressed exclusively by HEVs. Treatment with these MECA-79–anti-CD40L-NPs markedly delayed the onset of heart allograft rejection and increased the presence of Tregs. Finally, combined MECA-79–anti-CD40L-NPs and rapamycin treatment resulted in markedly longer allograft survival than soluble anti-CD40L and rapamycin. These data demonstrate that FRCs are critical to facilitating costimulatory blockade. LN-targeted nanodelivery of anti-CD40L could effectively promote heart allograft acceptance.

## Introduction

Lymph nodes (LNs) are integral sites at which the alloimmune response is mounted following transplantation. Our group has shown that LNs also function as sites of immune regulation by performing critical roles in the establishment of immune tolerance (1–6). The compartmentalized microarchitecture of the LN is crucial to the generation of effective alloimmune responses, due to the activity of specialized stromal cells called fibroblastic reticular cells (FRCs) and LN-specific segments of microvasculature (7). FRCs are mesenchymal cells that contribute to steering the alloimmune response, and they also create the matrix fibers that preserve the structural integrity of the LN (8, 9). FRCs have a characteristic cell signature defined by the presence of podoplanin (PDPN) and absence of the vascular marker CD31 or the leukocyte marker CD45 (9, 10). FRCs produce chemokines such as CCL19 that attract T cells, which enter the LN via specialized microvasculature known as high endothelial venules (HEVs) (11). Inside the LN, T cells navigate by attaching to the fibrous network created by FRCs, where they may interact with dendritic cells (DCs) or exit the LN (12, 13). In this manner, the activity of FRCs is integral to facilitating the contact between T cells and DCs, an interplay that is fundamental to the process of allorecognition.

Costimulation is a critical step in T cell activation, and costimulatory blockade at the time of T cell activation leads to T cell anergy and allograft tolerance (14–16). The immunosuppressive agents used currently can cause serious complications, including infection, malignancy, metabolic disorders, microvascular toxicity, and a higher death rate in transplant recipients (17–24). Therefore, the improvement of long-term transplant outcomes through the development of safer and more effective immunosuppressive strategies is a major unmet medical need (17, 21, 25–31). The CD40/CD40L costimulatory pathway plays a central role in T cell–mediated activation and maturation of DCs (32, 33). Blockade of the CD40/CD40L pathway induces the expansion of antigen-specific regulatory T cells (Tregs) (34–36). Given its role in controlling several arms of the adaptive immune response, the CD40/CD40L pathway represents a promising therapeutic target for the prevention of transplant rejection (37). However, there is a need for strategies to improve the efficacy of CD40L blockade to control T cell allorecognition and activation more effectively (38).

Here, we sought to examine the role of FRCs of LNs in prolonging graft acceptance mediated by the costimulatory blockade agent anti-CD40L. We also developed a targeted method of delivering anti-CD40L to LNs to improve its tolerogenic effect.

## Results

### FRCs are critical in anti-CD40L–induced long-term heart allograft survival.

We first investigated the role of the LNs as a critical site for mediating tolerance induced by costimulatory blockade using anti-CD40L. Hearts from BALB/c mice were heterotopically transplanted into either wild-type C57BL/6 (WT) or LTβR-knockout C57BL/6 (LTβR-KO) recipients that lack LNs (39). Both recipients were treated with anti-CD40L (125 μg, twice/day, i.v., days 0–1 after transplantation). As shown in Supplemental Figure 1A (supplemental material available online with this article; https://doi.org/10.1172/JCI159672DS1), LTβR-KO recipients lacking LNs were unable to establish long-term allograft survival as compared with WT recipients (mean survival time [MST] was 54 days vs. >100 days). These data indicated that lack of LNs in recipients could abrogate the ability of anti-CD40L to establish long-term allograft survival.

**Figure 1:**
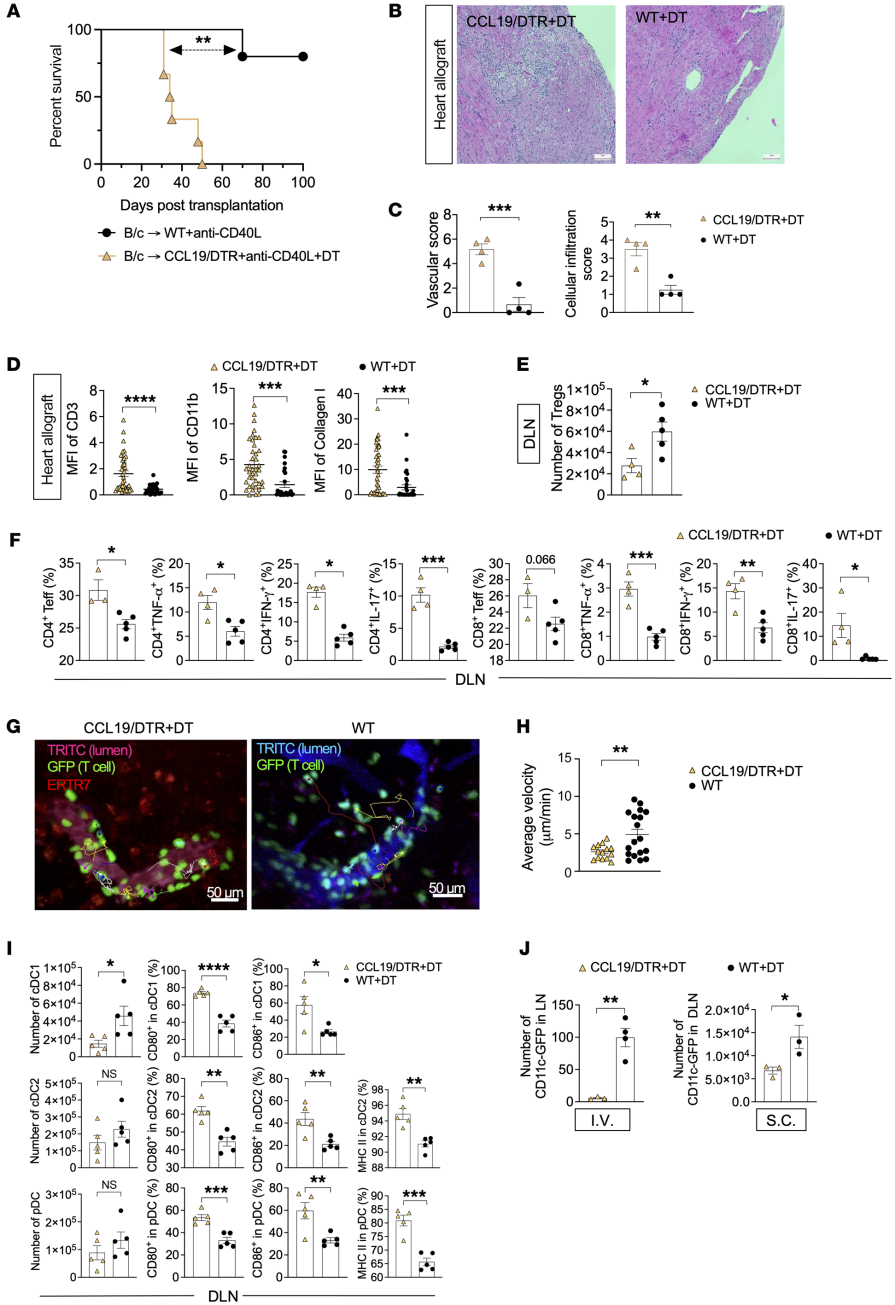
FRCs are critical for anti-CD40L–induced long-term heart allograft survival. (**A**) Comparison of heart allograft survival between WT (*n* = 5 mice/group, MST > 100 days) and CCL19/DTR recipients (*n* = 6 mice/group, MST = 34.5 days) of BALB/c (B/c) hearts treated with high-dose anti-CD40L and DT. Log-rank test for graft survival. (**B**) Representative light micrographs of H&E-stained heart allograft sections from WT and CCL19/DTR recipients on day 50 after heart transplantation. Scale bars: 100 μm. (**C**) Comparison of cellular infiltration and vascular damage of heart allografts in WT and CCL19/DTR recipients (*n* = 4 mice/group). (**D**) Comparison of MFI of CD3^+^ cells, CD11b^+^ cells, and collagen I^+^ cells in heart allografts from WT and CCL19/DTR recipients (*n* = 4 mice/group). (**E**) Comparison of Treg numbers in DLNs from WT and CCL19/DTR recipients by flow cytometry (*n* = 4–5 mice/group). (**F**) Comparison between percentages of CD4^+^ Teffs, CD4^+^TNF-α^+^, CD4^+^IFN-γ^+^, CD4^+^IL-17^+^, CD8^+^ Teffs, CD8^+^TNF-α^+^, CD8^+^IFN-γ^+^, and CD8^+^IL-17^+^ cells in the DLNs of WT and CCL19/DTR recipients by flow cytometry (*n* = 4–5 mice/group). (**G**) Intravital imaging showed GFP^+^ T cells migrating around the HEVs in the DLNs of CCL19/DTR and WT skin allograft recipients. Scale bars: 50 μm. (**H**) Comparison of average velocity of T cells in the DLNs from WT and CCL19/DTR mice. (**I**) Comparison between numbers of type I conventional DCs (cDC1), type II conventional DCs (cDC2), and peripheral DCs (pDC) and percentages of CD80^+^ cDC1, CD86^+^ cDC1, CD80^+^ cDC2, CD86^+^ cDC2, MHC II^+^ cDC2, CD80^+^ pDC2, CD86^+^ pDC2, and MHC II^+^ pDC2 in the DLNs of WT and CCL19/DTR recipients by flow cytometry (*n* = 4–5 mice/group). (**J**) Comparison between numbers of CD11c-GFP cells in DLNs from WT and CCL19/DTR mice 2 hours after i.v. or s.c. injection (*n* = 3 mice/group). Student’s *t* test for 2-group comparisons. Data presented as mean ± SEM. *P < 0.05, **P < 0.01, ***P < 0.001, ****P < 0.0001.

Then, we examined the importance of FRCs in the mediation of tolerance by depleting FRCs in CCL19/DTR mice using diphtheria toxin (DT), as previously described (1, 11, 12, 40). Hearts from BABL/c mice were transplanted into either CCL19/DTR or WT recipient mice, and recipients were treated with anti-CD40L as well as DT around day 25 after transplantation. We found that depletion of FRCs in recipients abrogates the long-term effect of anti-CD40L (MST 34.5 days) in comparison with the WT recipients (MST >100 days) (Figure 1A).

Histologic examination by H&E staining revealed a more severe cellular infiltration and occluded vasculature in the CCL19/DTR+DT group, as compared with the WT+DT group (Figure 1, B and C). Immunofluorescent staining of heart allografts showed higher CD11b^+^ cells and CD3^+^ cell infiltrates in the heart allografts recovered from the CCL19/DTR+DT group, as compared with the WT+DT group (Figure 1D). As interstitial fibrosis is an important feature of chronic rejection, we stained the heart allograft for collagen I, and it revealed a higher density of collagen I in the CCL19/DTR+DT group when compared with the WT+DT group (Figure 1D).

We noticed a significantly higher number of Tregs (Figure 1E) and CD4^+^ cells (Supplemental Figure 1B) in draining LNs (DLNs) from WT recipients when compared with CCL19/DTR recipients. We also found a significantly lower percentage of CD4^+^ and CD8^+^ T effector cells (Teffs), TNF-α^+^, IFN-γ^+^, and IL-17^+^ cells in the WT group as compared with the FRC-depleted group in a mixed lymphocyte reaction (MLR) assay (Figure 1F). We also noticed a significantly lower percentage of CD4^+^TNF-α^+^, CD4^+^IFN-γ^+^, and CD4^+^IL-17^+^ cells and a lower percentage of CD8^+^IL-17^+^ cells in the spleens of the WT group than in the FRC-depleted group (Supplemental Figure 1C). As IL-10 signaling is required for suppression of Th17 cell–mediated inflammation (41), we were interested to quantify the CD4^+^IL-10^+^ cells, but no significant difference was found between the WT and FRC-depleted groups (data not shown).

Following these findings, we tested the hypothesis that depleting FRCs would markedly reduce the trafficking of T cells into the LN and their conversion to Tregs under anti-CD40L treatment (42). We performed intravital imaging of the DLNs from murine skin transplant recipients on day 8 after transplantation to monitor the T cell trafficking in both WT and CCL19/DTR+DT mice. As shown in Figure 1G, injected labeled T cells displayed impaired extravasation across the HEVs in CCL19/DTR+DT mice, as compared with the WT mice, in which most T cells were already extravasated. In the LN, T cell motility is required for migration within the T cell zone and for making contacts with antigen-presenting DCs. After activation, motility permits the escape of T cells from the LNs, a process that is essential for the exertion of effector function (43, 44). The average velocity of the T cells was observed to be lower in the FRC-depleted DLNs, as compared with the WT DLNs (Figure 1H). As anti-CD40L mediates its action through the interaction between T cells and DCs, we examined the anatomical position of these cells. We used CD11c-GFP mice as recipients of skin transplants from BABL/c mice to track DCs (45). T cells from naive WT spleens were labeled with CytoTrace Red CMTPX and injected into CD11c-GFP recipient mice immediately before intravital imaging. FRC-built matrix was labeled with an anti–ERTR7 antibody conjugated with Alexa Fluor 647 and injected 12 hours before imaging. FRCs, DCs, and T cells were visualized by intravital imaging, and we found that T cells that interacted with CD11c^+^ DCs were mostly in the vicinity of FRCs (Supplemental Figure 1D).

We then examined the DLNs from WT and CCL19/DTR recipients for DC subtypes (46–48). As shown in Figure 1I, a reduction in the number of CD11c^+^ cells in the DLNs was observed in CCL19/DTR as compared with the WT group. The latter can contribute to fewer interactions with T cells using their CD40/40L interactions and subsequent Treg formation under anti-CD40L treatment. Further phenotyping DCs, we also found a higher percentage of DCs with less allogenicity (i.e., lower expression of positive costimulatory molecules) in the DLNs from WT recipients as compared with CCL19/DTR recipients. We were also interested in examining whether the reduction in CD11c^+^ cells was due to impaired trafficking into the LNs through afferent lymphatics or a decrease in migration through HEVs. As shown in Figure 1J, depletion of FRCs reduced DC migration to the LNs via both routes. We also found no significant difference in the number of DCs in the spleens (Supplemental Figure 1E). While the interaction between FRCs and DCs would require more complex studies using reporter mice, we found that depletion of CD11c^+^ DCs (using CD11c-DTR mice) abrogated the long-term effect of anti-CD40L in our murine model of heart transplantation (Supplemental Figure 1F). These findings suggest that interaction between FRCs and DCs might be critical in mediating immunoregulation by anti-CD40L.

### Anti-CD40L treatment altered the FRC population in LNs.

**Figure 2:**
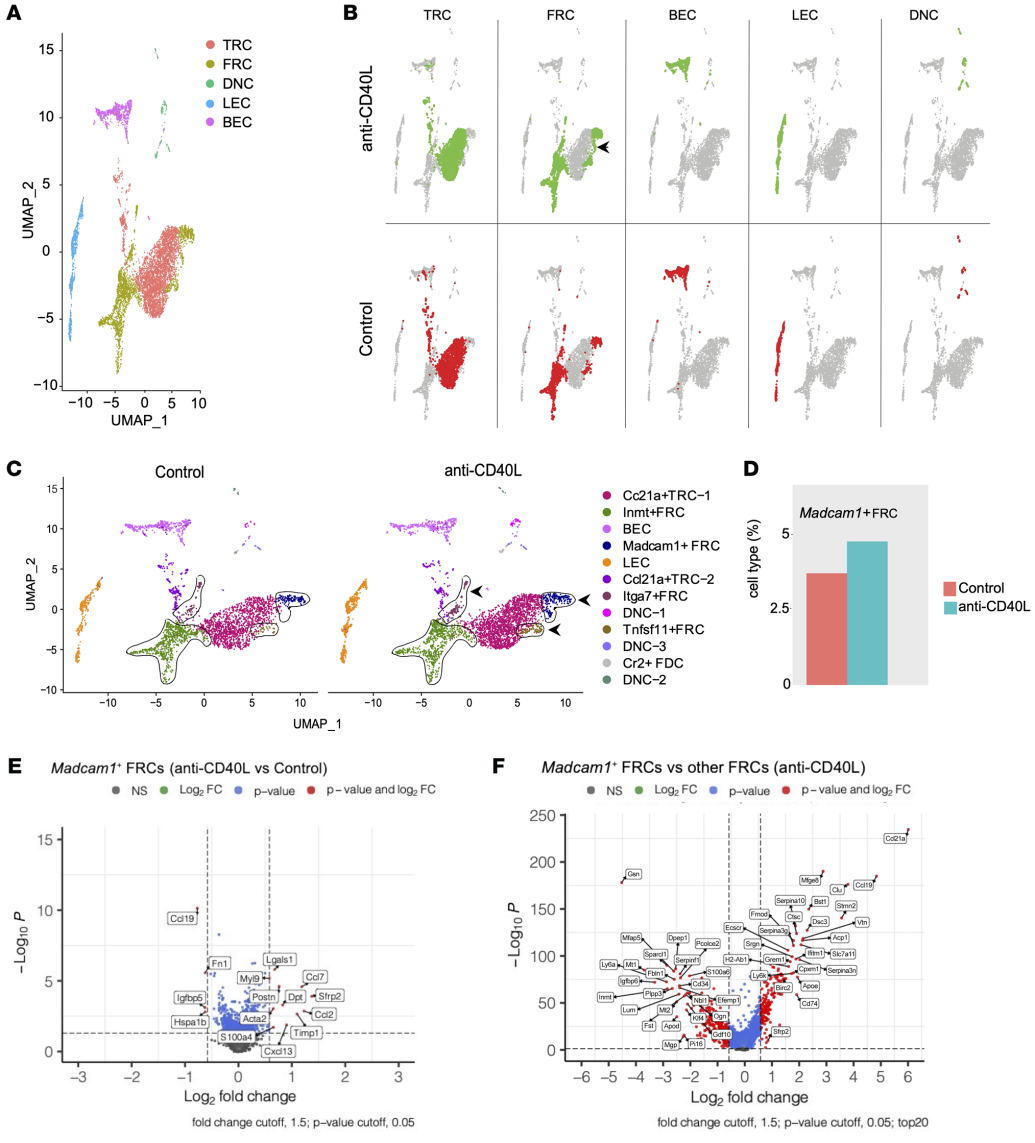
Anti-CD40L treatment alters the phenotype of FRCs. (**A**) Uniform manifold approximation and projection (UMAP) displays the stromal cell population map in LNs. (**B**) UMAP map from **A**, showing cell events by condition: anti-CD40L (top row) versus isotype control (bottom row). (**C**) UMAP visualization of clustering of different FRC populations, showing isotype control on left and anti-CD40L on right. The arrow shows the subset of FRCs that increase following anti-CD40L treatment. (**D**) Bar graph shows comparison of *Madcam1*^+^ FRC populations from **C**. (**E**) Volcano plot shows comparison of gene expression in *Madcam1^+^* FRCs between the anti-CD40L–treated group and isotype control–treated group. (**F**) Volcano plot showing gene expression in *Madcam1^+^* FRCs in comparison to other FRC subsets in the anti-CD40L–treated group.

The impact that immunomodulatory agents can exert on the phenotypes of FRC subsets in the LN can shape their immunoregulatory effects significantly. However, this concept remains understudied. Thus, we treated WT mice with anti-CD40L and collected the LNs for single-cell RNA sequencing (scRNA-seq) analysis of LN stromal cells (LNSCs). According to the expression of distinctive genes (49), the LNSC subclusters were classified into *Ccl21^+^* T reticular cells (TRCs), *N*-methyltransferase^+^ (*Inmt^+^*) FRCs, *Madcam1^+^* FRCs, *Itga7^+^* FRCs, *Tnfsf11^+^* FRCs, *Cr2^+^* follicular DCs (FDCs), *Pecam1^+^Pdpn^–^* blood endothelial cells (BECs), *Pecam1^+^Pdpn^+^* lymphatic endothelial cells (LECs), and *Pecam1^–^Pdpn^–^* double-negative cells (DNCs) (Figure 2, A and B). As compared with the control mice that received an isotype control antibody, treatment with anti-CD40L did not result in new LNSC subclusters (Figure 2, A and B). However, relative to controls, administration of anti-CD40L increased the *Madcam1^+^* FRCs (Figure 2, C and D). These results indicate that administration of anti-CD40L altered the LN FRC composition. Analysis of differential gene expression (DEG) among the subsets revealed that the *Madcam1*^+^ FRCs had increased expression of secreted frizzled-related protein 2 (*Sfrp2*) after anti-CD40L treatment (Figure 2E). *Sfrp2* expression exerts immunosuppressive effects in fibroblasts (50–53). *Madcam1*^+^ FRCs also demonstrated higher expression of *Ccl19* and *Ccl21* in comparison with other FRC subclasses (Figure 2F). Chemokines like CCL19 and CCL21 are critical for the recruitment of naive T cells to LNs, which again is important for the formation of Tregs under anti-CD40L therapy (54–57).

### FRCs exert tolerogenic regulation on CD4^+^ T cells.

**Figure 3:**
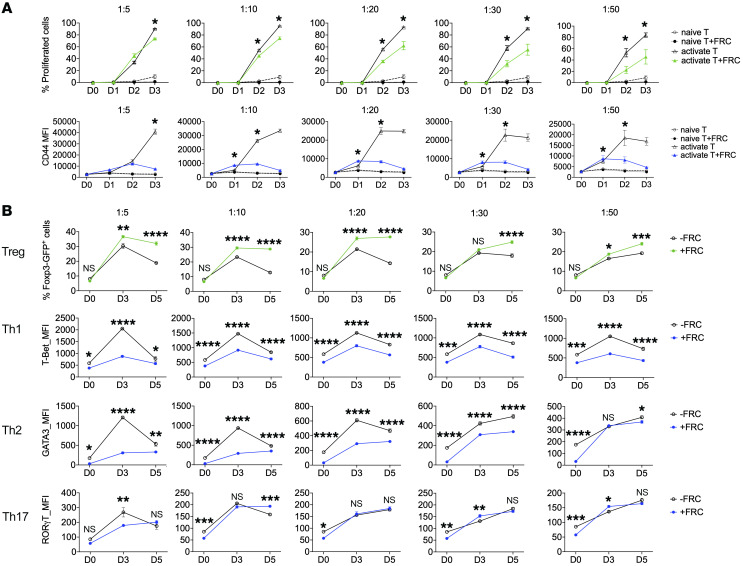
FRCs exert tolerogenic regulation on CD4^+^ T cells. (**A**) Analysis of naive versus activated T cells with/without FRC coculture at different time points. Flow cytometric analysis demonstrating MFI of different T cell subtypes on days 0, 1, 2, and 3 following coculture with FRCs in complete T cell medium supplemented with anti-CD28 and anti-CD3. FRCs suppressed T cell proliferation and activation as analyzed by flow cytometry on days 0, 1, 2, and 3. (**B**) CD4^+^ T cell differentiation in the presence or absence of FRCs was analyzed along with different FRC and T cell ratios by flow cytometric assay on days 0, 3, and 5. In the presence of FRCs, a higher percentage of Foxp3-GFP^+^ cells and lower MFI of Th1, Th2, and Th17 cells were found. Two-way ANOVA with Sidak’s multiple comparisons test for multiple comparisons of each group. Data presented as mean ± SEM. *P < 0.05, **P < 0.01, ***P < 0.001, ****P < 0.0001.

To evaluate the influences of FRCs on T cells, we cocultured FRC cell lines with naive CD4^+^ T cells and assessed CD4^+^ T cell activation, proliferation, and differentiation (58). During 3 days of coculturing in complete T cell medium supplemented with anti-CD28 and anti-CD3, FRCs suppressed T cell proliferation and activation (Figure 3A). Next, we examined the influence of FRCs on CD4^+^ T cell differentiation during coculturing. FRCs promoted CD4^+^ T cell differentiation to Tregs, but they suppressed CD4^+^ T cell differentiation to Teffs, including Th1, Th2, and Th17 cells (Figure 3B). These results indicated that FRCs were favorable for CD4^+^ T cell differentiation to Tregs while inhibiting T cell proliferation and activation. Given the critical role Tregs play in mediating the effects of anti-CD40L, FRCs may promote the tolerogenic effects of anti-CD40L through formation of Tregs (59).

We also examined the expression of Madcam1 on FRCs by flow cytometry. As shown in Supplemental Figure 2A, Madcam1 was highly expressed by FRCs. To examine the mechanism by which FRCs might promote formation of Tregs, we examined the expression of immunoregulatory cytokines produced by FRCs as well. As shown in Supplemental Figure 2B, FRCs expressed high levels of IL-10, IL-33, TGF-β, and PD-L1, which are important for the promotion of Treg formation (60, 61). However, CD40 expression was low in FRCs (Supplemental Figure 2C). To address any intrinsic response by FRCs to CD40L, we performed expression studies of genes for immune regulation and matrix fibers in FRCs, following treatment with anti-CD40L in vitro. No difference was found in the gene expression of *Ido*, *Tgfb*, *Arginase1*, *Pdl1*, and *Col1a1* between the anti-CD40L–treated and untreated groups (Supplemental Figure 2D).

### Synthesis and characterization of MECA-79 surface-coated anti-CD40L NPs.

**Figure 4:**
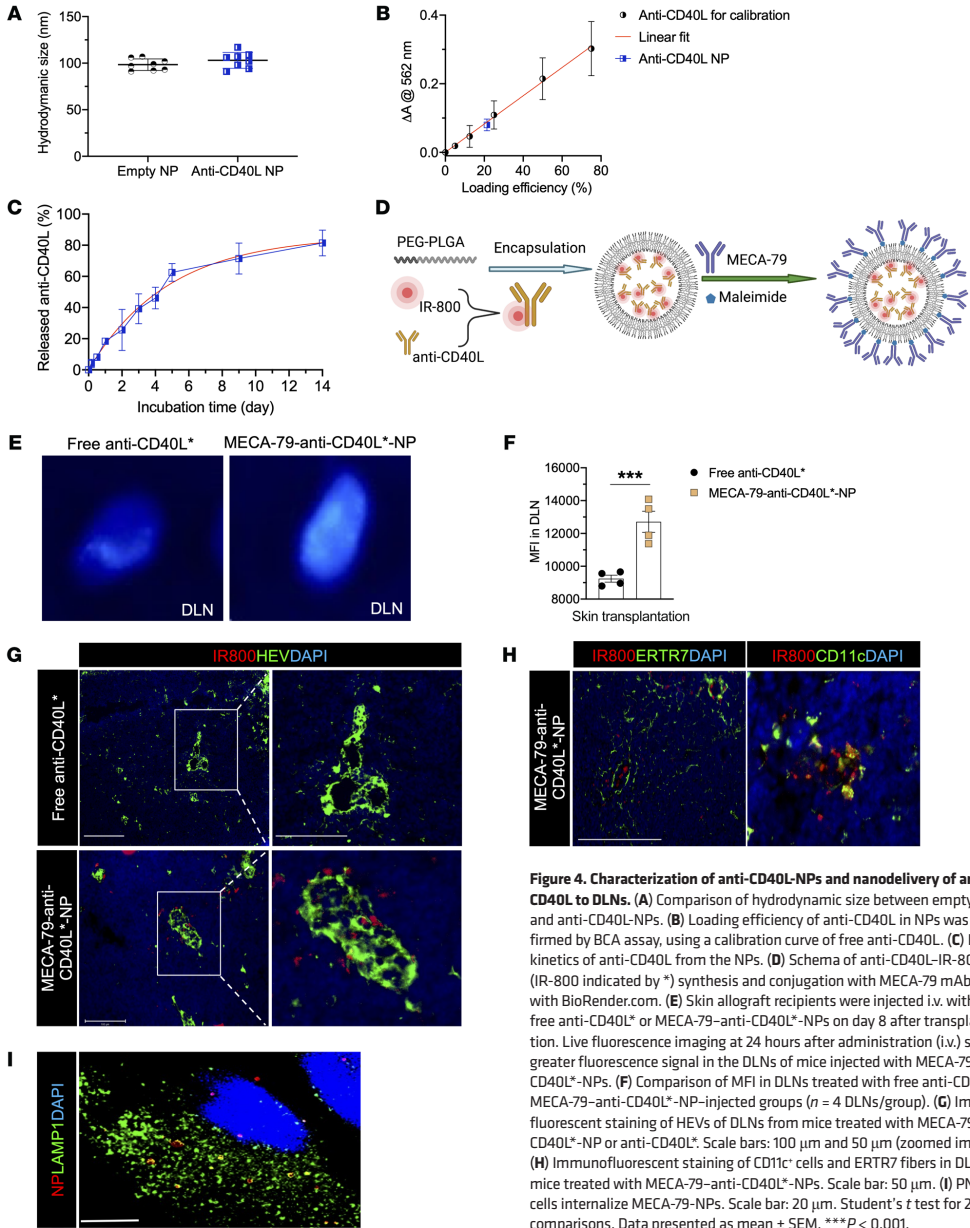
Characterization of anti-CD40L-NPs and nanodelivery of anti-CD40L to DLNs. (**A**) Comparison of hydrodynamic size between empty NPs and anti-CD40L-NPs. (**B**) Loading efficiency of anti-CD40L in NPs was confirmed by BCA assay, using a calibration curve of free anti-CD40L. (**C**) Release kinetics of anti-CD40L from the NPs. (**D**) Schema of anti-CD40L–IR-800 (IR-800 indicated by *) synthesis and conjugation with MECA-79 mAb. Created with BioRender.com. (**E**) Skin allograft recipients were injected i.v. with either free anti-CD40L* or MECA-79–anti-CD40L*-NPs on day 8 after transplantation. Live fluorescence imaging at 24 hours after administration (i.v.) showed greater fluorescence signal in the DLNs of mice injected with MECA-79–anti-CD40L*-NPs. (**F**) Comparison of MFI in DLNs treated with free anti-CD40L*– or MECA-79–anti-CD40L*-NP–injected groups (*n* = 4 DLNs/group). (**G**) Immunofluorescent staining of HEVs of DLNs from mice treated with MECA-79–anti-CD40L*-NP or anti-CD40L*. Scale bars: 100 μm and 50 μm (zoomed images). (**H**) Immunofluorescent staining of CD11c^+^ cells and ERTR7 fibers in DLNs of mice treated with MECA-79–anti-CD40L*-NPs. Scale bar: 50 μm. (**I**) PNAd^+^ CHO cells internalize MECA-79-NPs. Scale bar: 20 μm. Student’s *t* test for 2-group comparisons. Data presented as mean ± SEM. ***P < 0.001.

Poly(D,L-lactide-co-glycolide)–based (PLGA-based) nanoparticles (NPs) encapsulating anti-CD40L were prepared by a water/oil/water double emulsion method using ethyl acetate as an organic solvent, as previously described (3, 62, 63). MECA-79 monoclonal antibodies (mAbs) were reduced to generate sulfhydryl groups, which covalently bonded to terminal maleimide groups of the NPs to form MECA-79–anti-CD40L-NPs. The hydrodynamic size of these NPs did not change significantly following the encapsulation in comparison with empty NPs (Figure 4A). The loading efficiency of anti-CD40L in the NPs was approximately 21% (Figure 4B). Release of anti-CD40L from the NPs was sustained over 2 weeks in our in vitro kinetic assay (Figure 4C).

### Nanodelivery of anti-CD40L to DLNs.

We first examined whether MECA-79–anti-CD40L-NPs increase the delivery of anti-CD40L to the DLNs. We labeled anti-CD40L with IR-800 dye using *N*-hydroxysuccinimide (NHS) chemistry (referred as anti-CD40L*) and encapsulated inside MECA-79-NPs (Figure 4D) (3, 63, 64). Free anti-CD40L* and MECA-79-anti-CD40L*-NPs were injected i.v. on day 8 after skin transplantation. Pharmacokinetic studies were carried out 24 hours later. As shown in Figure 4E, we detected a higher fluorescent signal in the DLNs of mice injected with MECA-79–anti-CD40L*-NPs, as compared with those injected with free anti-CD40L*. The mean fluorescence intensity (MFI) of anti-CD40L* in the DLNs was significantly higher in MECA-79–anti-CD40L*-NP–injected mice than in the free anti-CD40L*–injected mice (Figure 4F). We also compared the MFI of anti-CD40L* between the DLNs and nondraining LNs (NDLNs) from the MECA79–anti-CD40L*-NP–injected group. As shown in Supplemental Figure 3A, the MFI of anti-CD40L* was significantly higher in the DLNs than NDLNs. We also found significantly higher MFI of anti-CD40L* in the kidney, liver, and spleen in the MECA-79–anti-CD40L*-NP–treated group (Supplemental Figure 3B). Immunofluorescent staining of the DLNs from MECA-79–anti-CD40L*-NP–injected mice indicated that anti-CD40L was present in the vicinity of the HEVs (Figure 4G). A portion of anti-CD40L* appeared to be internalized by CD11c^+^ cells (Figure 4H). By using fluorescence microscopy, MECA-79-NP entered peripheral node addressin^+^ (PNAd^+^) CHO cells, as deduced from the colocalization of NP fluorescence (red) and lysosomes (green) stained with an anti–lysosome-associated membrane protein 1 (anti-LAMP1) antibody (Figure 4I). Alex Fluor 594–labeled MECA-79-NPs were then injected into either CD11c-GFP or HEV-GFP mice (which express GFP only in HEVs of LNs and in intestinal villi; see Methods) and DLNs were subjected to intravital imaging, which revealed that MECA-79-NPs were found in the vicinity of the HEVs in DLNs (Supplemental Figure 3C) and were also endocytosed by CD11c^+^ cells residing within the interstitium of DLNs (Supplemental Figure 3D).

Similar results were found in the DLNs of mice that had undergone heart transplantation using free anti-CD40L* and MECA-79–anti-CD40L*-NPs applied i.v. to recipient mice. MFI of anti-CD40L* in the DLNs was significantly higher in MECA-79–anti-CD40L*-NP–injected mice than in the free anti-CD40L*–injected mice (Supplemental Figure 3, E and F).

To visualize the microanatomical localization of MECA79-NPs in the HEVs, immunofluorescent staining of LNs was carried out at both early and late time points after i.v. administration of MECA-79-NP–Alexa Fluor 594. As shown in Supplemental Figure 3G, 1 hour following the injection of MECA-79-NP–Alexa Fluor 594, labeled NPs were located on the apical side of the HEVs, whereas most of the NPs were found within the parenchyma of the LNs 24 hours after injection.

Exocytosis via microtubule activity has been reported to play a role in transporting NPs outside of cells (65–67). Therefore, we used the microtubule inhibitor colchicine to gauge the importance of microtubule activity to the exocytosis of our NPs by using PNAd^+^ CHO cells (68, 69). The data showed that exocytosis of MECA-79-NPs was reduced following treatment with colchicine, indicating that these NPs are transported by microtubules to the cell membrane during this process (Supplemental Figure 1H).

### Treatment with MECA-79–anti-CD40L-NPs prolongs heart allograft survival.

**Figure 5:**
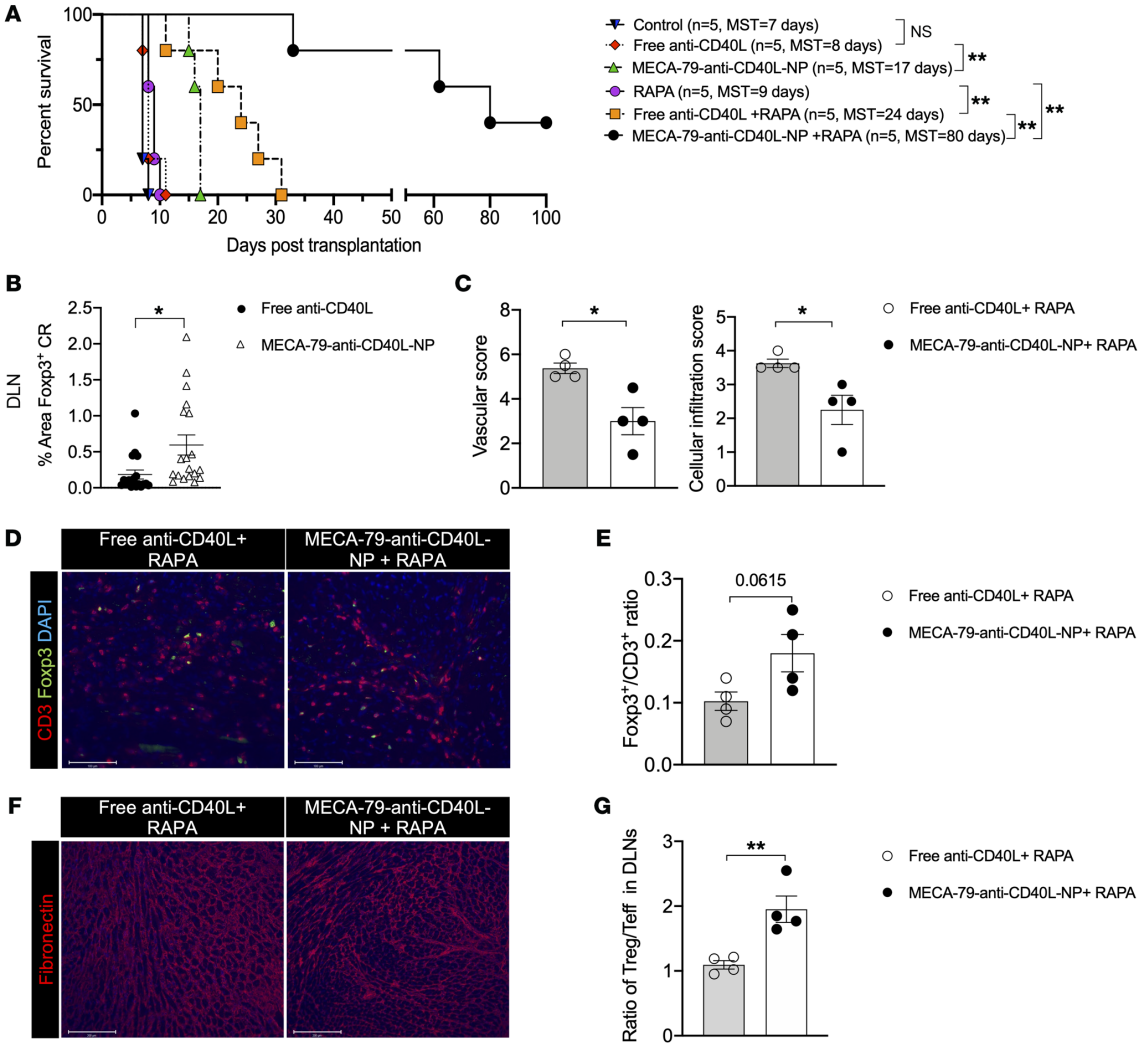
MECA-79–anti-CD40L-NPs alone or in combination with rapamycin prolongs heart allograft survival in mice. (**A**) Comparison of heart allograft survival between WT recipients of BALB/c hearts that were given no treatment, free anti-CD40L, or MECA-79–anti-CD40L-NPs (*n* = 5 mice/group; MST = 7 days vs. 8 days vs. 17 days, respectively); comparison of heart allograft survival between C57BL/6 recipients of BALB/c hearts that were treated with rapamycin (RAPA) (*n* = 5 mice/group, MST = 9 days), a combination of free anti-CD40L and RAPA (*n* = 5 mice/group, MST = 24 days), or a combination of MECA-79–anti-CD40L-NPs and RAPA (*n* = 5 mice/group, MST = 80 days). Log-rank test for graft survival. (**B**) Comparison of percentage of area in cortical area of Foxp3^+^ Tregs in DLNs by immunofluorescence. (**C**) Comparison of cellular infiltration and vascular damage between heart allografts in WT recipients following treatment with a combination of free anti-CD40L and RAPA or a combination of MECA-79–anti-CD40L-NPs and RAPA (*n* = 4 mice/group). (**D**) Representative florescence micrographs of CD3^+^ T cells and Foxp3^+^ Tregs in heart allograft sections of WT recipients. Scale bars: 100 μm. (**E**) Quantification of Foxp3^+^/CD3^+^ ratio in heart allografts by immunofluorescence. (**F**) Representative fluorescence micrographs of fibronectin staining in heart allograft sections of WT recipients. Scale bars: 100 μm. (**G**) Comparison of the Treg/Teff ratio in DLNs by flow cytometry. Student’s *t* test for 2-group comparisons. Data presented as mean ± SEM. *P < 0.05; **P < 0.01.

BALB/c mice hearts were transplanted into WT C57BL/6 recipient mice. Recipient mice were untreated (control) or treated with a low dose of free anti-CD40L or MECA-79–anti-CD40L-NPs from day –1 (before) to day 3 after transplantation (anti-CD40L doses were 9 μg/day, i.v., 5 days). We observed a significant prolongation of cardiac transplant survival following treatment with MECA-79–anti-CD40L-NPs (MSTs of untreated control recipient, free anti-CD40L, and MECA-79–anti-CD40L-NP heart allografts were 7, 8, and 17 days, respectively, *n* = 5 mice per group) (Figure 5A). These data indicate that targeted delivery of anti-CD40L to LNs prolongs its efficacy in allograft survival. Analysis of heart allografts from these mice revealed moderate to severe cellular infiltration and occluded vasculature in the free anti-CD40L group, while allografts from the MECA-79–anti-CD40L-NP–treated group contained much lower cellular infiltration and more intact vasculature (Supplemental Figure 4, A and B).

Immunofluorescent staining demonstrated that the DLNs in the MECA-79–anti-CD40L-NP–treated group contained significantly more Foxp3^+^ cells than those from the free anti-CD40L group (Figure 5B). As shown in Supplemental Figure 4C, MECA-79–anti-CD40L-NPs significantly suppressed the proliferation of T cells in an MLR assay as well.

### Combination of treatment with MECA-79–anti-CD40L-NPs and rapamycin induces long-term heart allograft survival.

After determining that MECA-79–anti-CD40L-NPs prolonged allograft survival, we wanted to test a potential synergistic effect with rapamycin (RAPA) with the capacity to not only suppress alloreactive T cells but also promote Treg formation (70, 71). Hearts from BALB/c mice were transplanted into WT recipients that were treated with either RAPA alone or combined with free anti-CD40L or MECA-79–anti-CD40L-NPs. MECA-79–anti-CD40L-NPs synergized with the immunoregulatory function of RAPA and extended the mean survival of heart allografts in comparison with the mice that received free anti-CD40L and RAPA (Figure 5A). We also found that depletion of FRCs abrogated the synergistic effects of RAPA and anti-CD40L in promoting acceptance of heart allografts (MST = 14 days, *n* = 4 mice per group).

Next, this experiment was repeated, but the mice were euthanized 21 days after transplantation, and heart allografts were examined to assess transplant rejection. H&E staining revealed moderate to severe injury of the heart allografts, with more severe cellular infiltration and occluded vasculature in the group treated with free anti-CD40L and RAPA, as compared with the group treated with MECA-79–anti-CD40L-NPs and RAPA (Supplemental Figure 4D). Pathologic scoring revealed significantly lower cellular and vascular injury in the MECA-79–anti-CD40L-NP+RAPA group (Figure 5C). Immunofluorescent staining of heart allografts revealed a higher ratio between Foxp3^+^ and CD3^+^ T cells in the MECA-79–anti-CD40L-NP+RAPA treatment group, as compared with the free anti-CD40L+RAPA group (Figure 5, D and E). The heart allografts also contained a lower density of fibronectin fibers in the MECA-79–anti-CD40L-NP+RAPA treatment group, as compared with the free anti-CD40L+RAPA group (Figure 5F). We also found that the ratio between Tregs and Teffs was significantly higher in the DLNs of the MECA-79–anti-CD40L-NP+RAPA group, as compared with the free anti-CD40L+RAPA group (Figure 5G). These studies demonstrate a synergistic effect between targeted delivery of the costimulatory-blocking anti-CD40L and RAPA.

## Discussion

The LN plays an important role not just in the formation of alloimmunity, but also in the formation of Tregs and immune regulation (72–75). The activities of FRCs are essential for maintenance of the compartmentalized microarchitecture of the LN, crucial to the generation of effective alloimmune responses as well as in mediating immune regulation (1, 8–10). FRCs also produce chemokines that attract T cells, which enter the LN via specialized microvasculature known as HEVs (11). The homing of these naive T cells to the LN is a prerequisite to the formation of Tregs, influenced by the presence of agents that promote immune regulation, including anti-CD40L (76–78). Once extravasated across HEVs, T cells navigate by attaching to the fibrous network and conduits created by FRCs, where they may interact with DCs, intercellular contact that is critical for the formation of Tregs through the effects of anti-CD40L within the LN (12, 77, 78).

Recent studies have identified various subclasses of FRCs that comprise the stromal compartments within the different regions of the LN. FRCs in the LN paracortex are important for supporting the interactions between DCs and T cells (7, 79). FRCs provide this support in several ways, including maintenance of HEV integrity, promotion of entry of naive T cells into the LN, and generation of a stromal compartment that supports the mechanical interaction between DCs and T cells (7, 79–81).

Costimulation is a critical step in T cell activation, and costimulatory blockade at the time of T cell activation leads to T cell anergy and allograft tolerance (14, 16, 82). Progress has been made in developing novel costimulatory blockade agents that target other pathways, including the promising CD40/CD40L that has been recently used in pig-to-primate cardiac xenograft models (83). Nanomedicines permit the direct delivery of a therapeutic payload to its target site, a phenomenon that augments its efficacy while limiting its off-target toxicity (84–88). However, this enticing potential of nanomedicine has not yet been fully developed in the field of transplantation (89–91).

Our data showing that the immunoregulatory effects of anti-CD40L were impaired in mice that lack LNs suggest that the mechanisms of tolerance induction occur in part within the microenvironment of the LN. The spleen may also play an important role here, as NF-κB–inducing kinase–KO and LTβR-KO mice can have a disorganized spleen that could interfere with the immune regulation induced by anti-CD40L (39, 92, 93). Interestingly, late depletion of FRCs also significantly abrogated long-term acceptance mediated by anti-CD40L. As the mechanism of action by anti-CD40L requires interaction between DCs and T cells, we were interested in identifying the location within the LN that most of this interaction occurs. Our intravital imaging revealed that many contacts between DCs and T cells occur immediately adjacent to the HEV. Depletion of FRCs could abrogate tolerance by interfering with the production of the necessary stromal fibers for this interaction between DCs and T cells that promotes Treg formation under costimulatory blockade (42). Depletion of FRCs could also impair homing of naive T cells to the LN. Intriguingly, our intravital imaging studies show that T cells are sequestered within the lumens of the HEVs following FRC depletion. We also noticed a decrease in DC populations in the FRC-depleted groups. Our trafficking studies of DCs suggested that depletion of FRCs impairs homing of DCs to LNs via both afferent lymphatics and across HEVs. Given the low expression of CD40 on FRCs and lack of intrinsic effects on FRCs with the anti-CD40L treatment, we thought that DCs play an important indirect role in mediating immune regulation controlled by FRCs under anti-CD40L treatment. In the same line of thinking, depletion of DCs abrogated the immunoregulatory effect of anti-CD40L in prolonging heart allograft survival.

Coculturing FRCs with T cells induced a shift in phenotype toward Tregs and a reduction in Th1, Th2, and Th17 classes. The impact of FRCs on T cells could be produced via secretion of a wide range of secretory immunoregulatory molecules, such as indoleamine 2,3-dioxygenase and prostaglandin E, or via expression of inhibitory surface molecules (12, 94). Our data showed that FRCs express high levels of IL-10, IL-33, TGF-β, and PD-L1, which are widely recognized immunoregulatory molecules (41, 95–97). These pathways need to be studied further in detail, using conditional knockout mouse strains to pinpoint their relative importance in organ transplantation. One very important but completely understudied topic is the impact that immunosuppressive agents may exert directory or indirectly (via DC–T cell interactions) on the phenotype of FRCs. Our scRNA-seq study showed that anti-CD40L treatment could potentially shift FRCs toward a more immunosuppressive phenotype. The expression of the *Sfrp2* gene was enriched in the *Madcam1^+^* FRC population of the anti-CD40L–treated group. SFRP2 belongs to the family of secreted frizzled-related proteins that interact with Wnt protein (98–100). SFRP2 has been shown to promote immunosuppressive-type immune infiltrates into tumors (101, 102). TIMP1, encoded by another enriched gene in the anti-CD40L–treated group, was found to suppress cytotoxic T cells as well (103). Future studies to query the transcriptomes of FRC subclasses under costimulatory blockade at various stages after transplantation would increase our understanding of how these therapeutics change the milieu of the LN. There is a need for more in-depth single-cell genomics of both DCs and FRCs in the DLN following treatment with anti-CD40L to further pinpoint the relative role of each cell type, such as ligand-receptor informatics analysis between cell subsets. The results of such studies will then direct us to what other conditional Cre-Lox strains will be informative to elucidate specific molecular mechanisms of cell activities and crosstalk. FRC mapping strategies would also permit the examination of the role of DCs and potential growth factors in transforming undifferentiated FRCs to the Madcam1^+^ subset. Examining the Madcam1^+^ cells in the spleen and the cells that drive immune regulation in both LNs and spleen are also important future endeavors.

A key issue that we have begun to address is to assess the effect of selective delivery of anti-CD40L to the LNs on the prolongation of heart allograft survival. Targeted drug delivery to the LN creates a plethora of applications to shift the microenvironment of the LN toward a tolerogenic microenvironment (1, 3, 104).

HEVs are extremely specialized vessels present exclusively in the LN that function as gateways for the entry of T cells. HEVs express a series of PNAd isoforms (105–109). MECA-79 is a mAb that recognizes all PNAd molecules in the HEVs (110–115). HEVs express PNAd on both their luminal and abluminal sides (115, 116). The extensive distribution of PNAd on the tip of the microvilli on the luminal membrane of the HEV has been reported to be important for interaction with L-selectin in inducing the rolling of lymphocytes, the first step in their transendothelial migration (117, 118). Depletion of FRC and reduction in CCL19 could be the main contributors to impairment of T cell trafficking noted in our live imaging of LNs of FRC-depleted mice.

Targeted delivery of anti-CD40L to the LNs via MECA-79–anti-CD40L-NPs significantly prolongs heart allograft survival, as compared with free anti-CD40L treatment. Moreover, treatment of transplant recipients with MECA-79–anti-CD40L-NPs combined with RAPA induces long-term heart allograft survival in comparison with free anti-CD40L combined with RAPA.

PLGA is widely used for synthesis of NPs, as it can be engineered and characterized readily and exhibits a good safety profile (119–127). PLGA NPs with diameters of approximately 100 nm have demonstrated superior biocompatibility and lower uptake by macrophages, as compared with larger NPs (128). Our live imaging shows that the early interaction occurs at the apical site of HEVs and their subsequent internalization into HEV cells from where these NPs are exocytosed into LN stroma near the peri-HEV environment using microtubules.

Furthermore, antibody conjugation through maleimide chemistry is currently the most studied process for the development of therapeutic platforms, and it is clinically approved (129, 130). An important point here is the fact that PNAd is constitutively expressed by the HEV vasculature in all LNs. Therefore, the idea that our platform may lead to the delivery of costimulatory blockade to all LNs and may result in generalized immunosuppression could raise concern. We believe because this delivery permits a reduction in the dosage of immunosuppressive drugs, its potential benefits outweigh this risk. Furthermore, as we have shown previously, the DLN has a much higher density of HEVs, PNAd expression, and blood flow than NDLNs, contributing to higher concentration of the drug within the DLN (3–6).

In summary, our understanding of mechanisms by which LNs determine the fate of alloimmune responses has evolved markedly following prior advances that have been made to understand more deeply the functions of specific cellular and stromal components of the LN (7, 79, 81, 131). The data from these studies could lay the groundwork to develop innovative therapeutic strategies aimed at manipulating the microenvironment within LNs. This provides a unique opportunity to direct the alloimmune response following transplantation toward tolerance.

## Methods

### Mice.

Seven- to 8-week-old WT C57BL/6J (stock 00064), BALB/cByJ (stock 001026), B6.Cg-Tg(Chst4-EGFP)23Nrud/J (referred to as HEV-GFP mice; stock 022787), C57BL/6-Gt (ROSA)26Sortm1 (HBEGF)Awai/J (C57BL/6 iDTR, referred to as DTR mice; stock 007900), B6.FVB-1700016L21RikTg (ItgaxDTR/EGFP)57Lan/J (CD11c-DTR/GFP, referred to as CD11c/DTR or CD11c-GFP mice; stock 004509), and C57BL/6-Tg (UBC-GFP)30Scha/J (referred to as UBC-GFP mice; stock 004353) were purchased from The Jackson Laboratory. CCL19^Cre^ mice were a gift from Shannon Turley at Genentech (South San Francisco, California, USA). LTβR-KO mice were obtained from Alexei Tumanov (The University of Texas Health Science Center, San Antonio, Texas, USA). CCL19^Cre^ mice were backcrossed with DTR mice to generate CCL19/DTR mice. Offspring were genotyped by PCR, according to the protocol from The Jackson Laboratory.

All male and female mice were housed in a specific pathogen–free animal facility. All experiments were performed with age- and sex-matched, 8- to 12-week-old mice.

### Mouse heterotopic cardiac transplantation and skin transplantation.

Vascularized intra-abdominal heterotopic transplantation of heart allografts and skin transplantation were performed using microsurgical techniques, as previously described (3, 63, 132–134). The status of the heart allograft was monitored daily by abdominal palpation. Rejection was defined as complete cessation of a palpable heartbeat and confirmed by direct visualization at laparotomy. Full-thickness skin grafts are usually rejected in 8 to 12 days. Intravital imaging and fluorescent imaging were performed in recipients on day 8 after transplantation.

### In vivo treatment protocol.

InVivoMab anti–mouse CD40L (anti-CD40L) was purchased from Bio X Cell. For the high-dose anti-CD40L groups, the mice received 250 μg anti-CD40L twice per day, i.v. on day –1 and day 0 after transplantation; for the low-dose anti-CD40L groups, the mice were treated with 9 μg anti-CD40L, daily, i.v. on day –1 to day 3 after transplantation. For depletion of FRCs, CCL19/DTR mice received 100 ng DT from *Corynebacterium* *diphtheriae* (MilliporeSigma) i.p*.* daily for 5 days. Mice received 25 μg of RAPA (Cayman Chemical) i.p*.* daily on days 0, 1, 2, and 3 after transplantations.

### scRNA-seq.

Three C57BL/6 mice at 12 weeks of age were treated (i.v.) with 250 μg anti-CD40L (Bio X Cell) for 1 day. The naive control mice received an isotype control antibody. Cell preparation and sequencing methods were described previously (135). The raw data have been deposited in the NCBI Gene Expression Omnibus database (GEO GSE213400 for anti-CD40L group; GSE202068 for control group) (135).

### scRNA-seq data analysis.

Seurat 3 was used for scRNA-seq analysis (136). First, genes in at least 3 cells were included in the analysis. Cells with fewer than 200 or more than 5500 unique genes and greater than 15% mitochondrial genes were excluded from downstream steps to exclude dead or doublet cells. After processing, 3329–4398 cells (out of 3422–4523 cells) were included for further analysis. The expression data were then log-transformed, normalized, and scaled using Seurat’s “ScaleData” function. Cells from the anti-CD40L–treated group were integrated with the isotype control–treated samples, using an anchor-based integration method implemented by Seurat, accounting for batch-effect correction. Thirty principal components of the integrated object were used for uniform manifold approximation and projection (UMAP) dimensionality reduction analysis, which then visualized the cells on a 2D UMAP plot. Differential expression analysis was done using model-based analysis of single-cell transcriptomics (MAST) (137). Only genes expressed in at least 10% of the cells in either group were considered differentially expressed if the adjusted *P* value was less than 0.05 and the absolute fold change was greater than 1.2.

### Coculture of FRCs and CD4^+^ T cells.

FRC lines were obtained from Sophie Acton (Stromal Immunology Group, Laboratory for Molecular Cell Biology, University College London, London, United Kingdom), and FRCs were cultured and maintained as previously described (138). Five thousand FRCs in 100 μL FRC media were placed in a 96-well plate. On the next day, CFSE^+^ CD4^+^ T cells were seeded at a concentration of 25,000–250,000 cells per well (FRC/T cell ratios of 1:5, 1:10, 1:20, 1:30, and 1:50) in 100 μL media plus 100 μL FRC media (DMEM, 1% penicillin/streptomycin, 10% FBS). For induction of T cell differentiation (Treg, Th1, Th2, Th17), anti-CD3/anti-CD28 Dynabeads (20 μL/10^6^ T cells; Thermo Fisher Scientific, 11456D) and cytokines were added and cultured at 37°C in 5% CO_2_. After 3 and 5 days, cells were harvested, and flow cytometry was performed. For CD4^+^ T cell activation, anti-CD3/anti-CD28 Dynabeads (20 μL/10^6^ T cells) and IL-2 (5 ng/mL) were added to the T cell media. To ensure adequate nutrition was provided to both cell types, 100 μL T cell media and 100 μL FRC media were used for coculture. Experiments were conducted in parallel in 5% CO_2_. After 1, 2, and 3 days, cells were harvested and flow cytometry was performed.

### In vivo fluorescent labeling.

T cells (2 × 10^7^ to 4 × 10^7^) were isolated from the spleens of UBC-GFP or WT C57BL/6 mice by magnetic-activated cell sorting (MACS) using EasySep Magnets (Stemcell Technologies), according to the manufacturer’s protocol. Higher than 98% purity of isolated T cells was confirmed by flow cytometric analysis using the pan–T cell marker CD3e. T cells from WT mice were labeled with CytoTrace Red CMTPX, according to the manufacturer’s protocol. To study trafficking of T cells in mice, T cells from UBC-GFP mice were injected into CCL19/DTR mice treated either with or without DT. To visualize the FRCs, CD11c^+^ cells, and T cells, CytoTrace Red CMTPX–labeled T cells were injected into CD11c-GFP mice on day 8 after skin transplantation. To visualize FRCs of a brachial LN, an anti-ERTR7 antibody conjugated with Alexa Fluor 647 (10 μg, 50 μL; Santa Cruz Biotechnology, sc-73355) was injected s.c. approximately 12 hours prior to imaging. To fluorescently label HEV lumens, tetramethylrhodamine isothiocyanate–Dextran (TRITC-Dextran; average mol wt 155,000 Da, 5 mg/mL, 100 μL; Sigma-Aldrich, T1287) dissolved in 1× PBS was i.v. injected, or HEV-GFP mice were used. To study trafficking of CD11c^+^ cells in mice, 2 × 10^7^ CD11c-GFP cells were s.c. or i.v. injected into WT and CCL19/DTR mice treated with DT.

### Mouse preparation and intravital imaging.

Mice were anesthetized by i.p. injection of a mixture of 100 mg/kg ketamine and 10 mg/kg xylazine. The brachial LN of the anesthetized mouse was surgically exposed by a small incision of skin and fascia at the brachial fossa. Intravital imaging was performed by using a laser scanning 2-photon microscope (IVM-MS2, IVIM Technology). GFP and TRITC were excited with a single-femtosecond pulse at 920 nm wavelength, and their emission was acquired simultaneously. Intravital images were obtained using a high numerical aperture water-immersion objective lens (CFI75 Apochromat 25XW, NA1.1; Nikon) with a field of view of 454 × 454 μm^2^. For time-lapse imaging, 25 to 30 sequential *Z*-stack images with a 2-μm axial interval were achieved at a 1-minute time interval for 0.5–1 hour.

### Flow cytometry.

Antibodies were purchased from BioLegend unless otherwise stated. Mouse antibodies (with their clone numbers) against the following proteins were used: CD4 (RM4-5), CD8a (53-6.7), CD25 (PC61), CD44 (IM7), CD62L (MEL-14), Foxp3 (MF-14), CD11b (M1/70), CD11c (N418), CD80 (16-10A1), CD86 (B7-2), IFN-γ (XMG1.2), IL-17 (TC11-18H10.1), MHC II (M5/114.15.2), PDPN (8.1.1), CD31 (390), Madcam1 (MECA-367), TGF-β (TW7-16B4), IL-10 (JES5-16E), PD-L1 (B7-H1), and CD40 (3/23). IL-33 (AF3626) was purchased from R&D Systems. For intracellular cytokine staining, cells were stimulated with phorbol 12-myristate 13-acetase (PMA, 50 ng/mL) and ionomycin (500 ng/mL) in combination with GolgiStop (BD Biosciences) for 4 hours, and then permeabilized and stained with necessary antibodies. Fluorescence was detected by a CYTEK AURORA (Cytek Biosciences) flow cytometer. Data were analyzed using FlowJo software.

### Preparation and characterization of anti-CD40L-NPs.

Anti-CD40L-NPs were synthesized as previously described (3). PEG-PLGA and maleimide-PEG-PLGA were dissolved in ethyl acetate. Anti-CD40L (clone MR-1, Bio X Cell) was diluted in PBS and added to the polymer mixture, and anti-CD40L-NPs were concentrated by centrifugation. The size and zeta potential of anti-CD40L-NPs were assessed and characterized using dynamic light scattering. The morphology of anti-CD40L-NPs was studied using transmission electron microscopy. The filtrate of the anti-CD40L-NPs was collected, and free anti-CD40L was quantified by BCA assay. The amount of anti-CD40L in the filtrate was quantified through comparison with a calibration curve of various concentrations of free anti-CD40L. To quantify the release profile of anti-CD40L from NPs, the anti-CD40L-NP solutions were incubated in triplicate at 37°C and assessed at defined time intervals. The samples were centrifuged at each time point using Amicon Ultra-15 centrifugal filter units (MWCO 10 kDa; Sigma-Aldrich) at 3000*g* for 15 minutes. The absorbances of the filtrate and anti-CD40L-NP suspension were then analyzed at 280 nm using a UV/VIS spectrophotometer. The amount of released anti-CD40L at each time point was quantified by comparing the absorbance at 280 nm with a calibration curve of various concentrations of anti-CD40L.

### Conjugation of MECA-79 to the surface of NPs.

MECA-79 mAb (NOVUS Biologicals, NB100-77673) was conjugated to the functional surface of NPs using thiol-maleimide chemistry. MECA-79 mAb (24 μL of 1.0 mg/mL solution) was pretreated with 24 μL TCEP (0.5 M, 15 minutes, room temperature) to cleave disulfide bonds of antibodies and was mixed immediately with the suspension of NPs. Maleimide groups on NPs bind covalently to the free thiols of the MECA-79 mAb. MECA-79-NPs were dialyzed (Sigma-Aldrich, Amicon, MWCO 10 kDa) to remove free antibodies and stored at 4°C before use.

### Synthesis of anti-CD40L–IR-800.

The IRDye 800CW NHS Ester (LI-COR; dissolved in DMSO) was added to anti-CD40L (molar ratio of dye to antibody, 1:10), mixed vigorously, and then incubated for 2 hours at room temperature. Excess IR-800 was removed by a desalting column (Thermo Fisher Scientific, Zeba, MWCO 7 kDa), and stored at 4°C before use.

### MLR assay.

LNs and spleens from WT and CCL19/DTR recipients were collected, splenocytes and lymphocytes were isolated, and 1 × 10^6^ cells were seeded in 96-well plates for each sample. CFSE working solution (5 μM) was incubated with the cells for 25 minutes at 37°C. DCs were isolated from BALB/c mouse spleens using CD11c MicroBeads UltraPure, 2 MS columns, and a MiniMACS Separator (all from Miltenyi Biotec). DCs (1 × 10^3^) were added to each sample, incubated for 3 days, then harvested for flow cytometry. Samples were stained for CD4, CD8, CD44, CD62L, TNF-α, IFN-γ, IL-17, IL-2, and IL-10. Irradiated donor (BALB/c) splenocytes (stimulator) and recipient (WT) splenocytes (responder) were added to each well of a 96-well round-bottom plate and incubated at 37°C for 2 days. ^3^H-thymidine (1 μCi) was added, and the plate was incubated for an additional 14 hours. The cells were transferred to a filter map by a Tomtec Harvester 96 cell harvester and analyzed by a 1450 MicroBeta TriLux microplate scintillation counter.

### Immunohistochemistry, immunofluorescent staining, and quantification.

Heart allografts and LNs were harvested at designated time points after transplantation. Heart allografts were fixed in formalin, embedded in a paraffin block, or preserved in optimal cutting temperature (OCT) compound (Tissue-Tek) and stored at –80°C. Samples were cut into 5-μm sections and stained with H&E. For immunofluorescent staining, sections were stained with conjugated or purified antibodies. Purified antibodies were detected using secondary antibodies. The antibodies included anti-CD11b (BioLegend, 101202), anti–collagen I (Abcam, ab34710), anti–Lyve-1 (Abcam, ab14917), anti-ERTR7 (Santa Cruz Biotechnology, sc73355), anti-fibronectin (Abcam, ab2413), anti-Foxp3 (BioLegend, 126401), and anti-CD3 (BioLegend, 100201). DAPI (VECTASHIELD, Vector Laboratories) was used to counterstain the cell nuclei. The stained tissue sections were visualized using an EVOS FL Auto 2 Imaging System (Thermo Fisher Scientific). Quantification was performed on 4 to 5 sections from at least 3 separate mice using Celleste (Invitrogen) and ImageJ (NIH, v1.8.0_112) image analysis software.

### Histological scoring and measurement.

Histological scoring of H&E-stained heart allografts was performed using a modified method from the International Society for Heart and Lung Transplantation (139, 140), as described previously (63, 133). Cellular infiltration was graded from 0 to 4. Vascular appearance was determined by a combination of vascular occlusion score and perivascular cellular infiltration. Vascular (artery) occlusion was scored from grade 0 to 3 for every artery. The sum of the vascular occlusion score and perivascular cellular infiltration score was designated as the vascular appearance score.

### Exocytosis study.

Adherent PNAd^+^ CHO cells were grown on 8-well plates (glass bottom) at a density of 2 × 10^4^/well. Cells were incubated with MECA79-NP–Alexa Fluor 594 for 2 hours. After washing, colchicine (100 μM) was incubated with cells for 2 hours. After removal of colchicine solution, we further incubated cells in fresh medium for 1 day and measured the fluorescence signal in the cells by EVOS fluorescence microscope. The NP signals from cells were quantified by ImageJ.

### Statistics.

All data were analyzed by Prism 9 (GraphPad Software) and are presented as mean ± SEM. Two-tailed Student’s *t* test and 2-way ANOVA were performed to determine significant differences among groups. Statistical evaluation of graft survival was analyzed by the Kaplan-Meier method, and the log-rank test was performed to determine the effect. Significance was defined as a *P* value of less than 0.05.

### Study approval.

All animal experiments were approved and performed in accordance with the guidelines and regulations of the IACUC of the Brigham and Women’s Hospital and Harvard Medical School in Boston, Massachusetts, USA. The study protocol was reviewed and approved by an Institutional Review Board at the Brigham and Women’s Hospital and was conducted in full conformance with the principles of the Declaration of Helsinki.

## Author contributions

JZ designed and performed experiments, analyzed and interpreted data, and drafted the manuscript. SJ designed and fabricated NPs, performed experiments, analyzed data, and wrote the part of Methods and Results. XL, LL, SNM, AM, GS, YH, VS, HZ, and YS performed experiments. BM, PK, JCM, PTS, SGT, SEA, and GCT helped with the study design. VK critically revised the manuscript. RA and JSB designed the study, interpreted the data, and critically revised and finalized the manuscript. The order of the co–first authors was decided by the time spent and scientific contribution to the paper.

## Supplementary Material

Supplemental data

## Version 1 12/15/2022

Electronic publication
