# Supplementary material for: Delivery of costimulatory blockade to lymph nodes promotes transplant acceptance in mice
Source: J Clin Invest. 2022 Dec 15;132(24):e159672. doi: 10.1172/JCI159672 (PMC9754003; doi:10.1172/JCI159672)
Supplement: Supplemental data [file jci-132-159672-s119.pdf]

Supplementary Figures and Figure legends:

Supplementary Figure.1

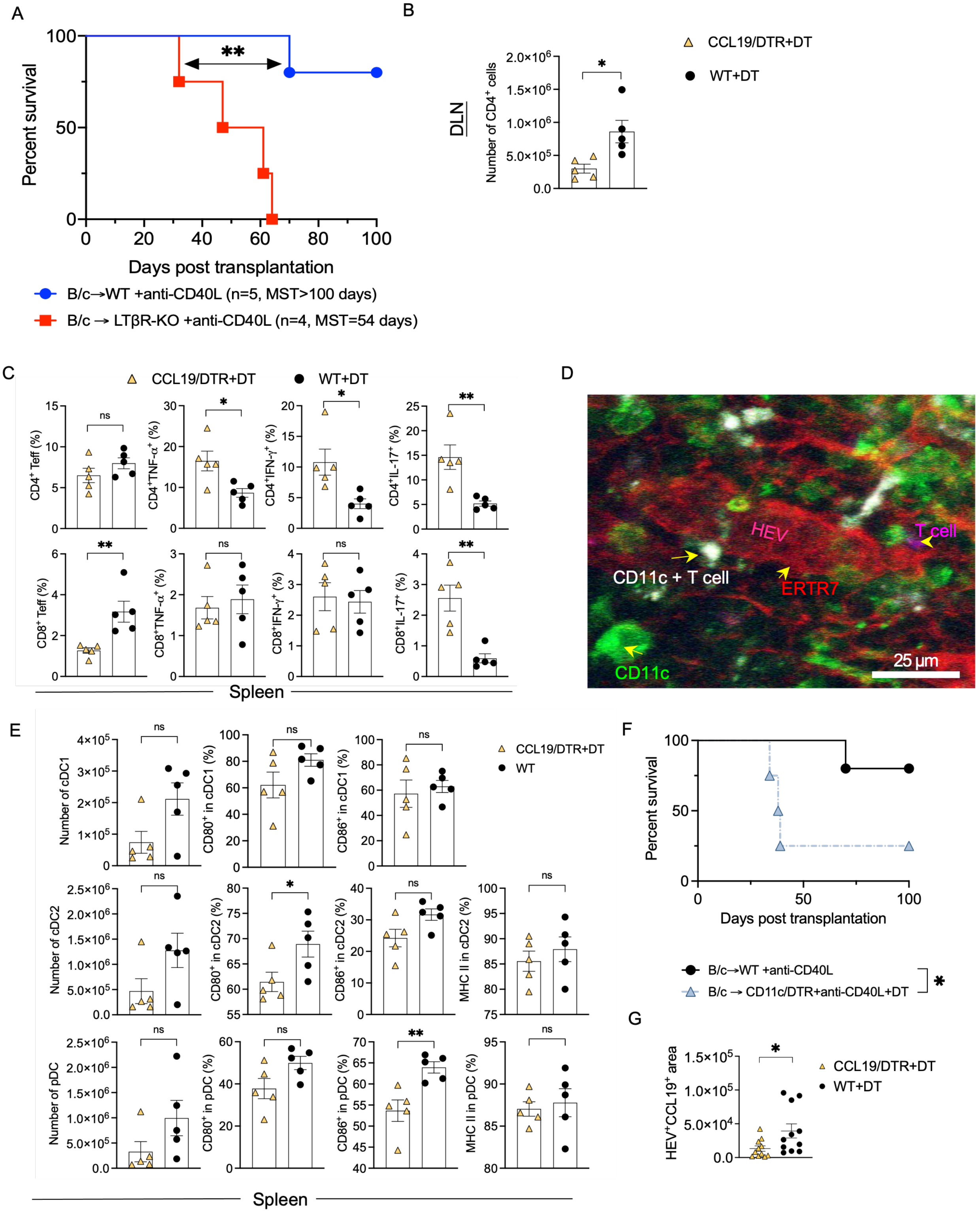

Supplementary Figure 1. *LNs are critical to anti-CD40L-induced long-term heart allograft survival.* (**A**) Comparison of heart allograft survival between WT C57BL/6 (n=5 mice/group, MST>100 days) and LTβR-KO C57BL/6 recipients (n=4 mice/group, MST=54 days) of BALB/c hearts treated with high dose anti-CD40L. (**B**) Comparison between numbers of $CD4^+$ cells in the DLNs of WT and CCL19/DTR recipients (n=4-5 mice/group). (**C**) Comparison between percentages of $CD4^+$Teff, $CD4^+TNF\alpha^+$, $CD4^+IFN\gamma^+$, $CD4^+IL\text{-}17^+$, $CD8^+$Teff, $CD8^+TNF\alpha^+$, $CD8^+IFN\gamma^+$ and $CD8^+IL\text{-}17^+$ cells in spleens of WT and CCL19/DTR recipients by flow cytometry (n=4-5 mice/group). (**D**) Anatomical position of FRCs, DCs, and T cells in the LN. (**E**) Comparison between numbers of cDC1, cDC2 and pDC and percentage of $CD80^+$cDC1, $CD86^+$cDC1, $CD80^+$cDC2, $CD86^+$cDC2, MHC II^+cDC2, $CD80^+$pDC2, $CD86^+$pDC2, MHC II^+pDC2 in spleens of WT and CCL19/DTR recipients by flow cytometry (n=4-5 mice/group). (**F**) Comparison between heart allograft survival of WT C57BL/6 (n=5 mice/group, MST>100 days) and CD11c/DTR C57BL/6 recipients (n=4 mice/group, MST=38.5 days) of BALB/c hearts treated with high dose anti-CD40L. (**G**) Comparison between HEV^+CCL19^+ areas in DLNs by IF (n=4-5 mice/group). Data presented as mean ± SEM, *p<0.05, ** p<0.01.

Supplementary Figure.2

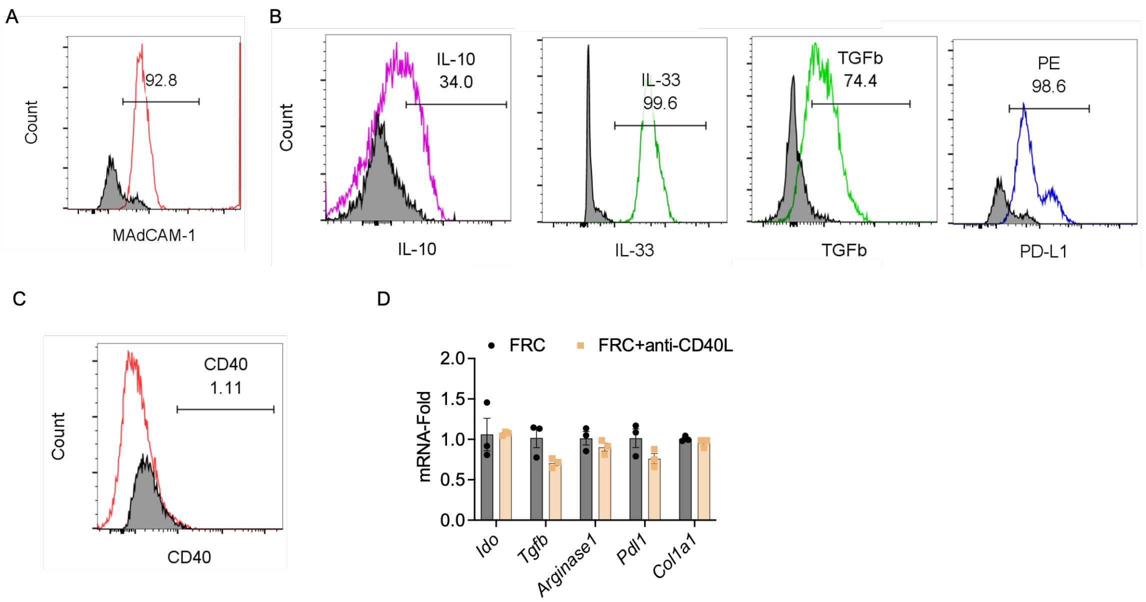

Supplementary Figure 2. (**A** and **B**) Madcam1, IL-10, IL-33, TGF-β and PD-L1 expression on FRC by flow cytometry. (**C**) CD40 expression on Madcam1+ FRC line by flow cytometry. (**D**) mRNA level of *Ido*, *Tgfb*, *Arginase1, Pdl1* and *Col1a1* from FRC and FRC+ anti-CD40L groups by qPCR. Data presented as mean ± SEM.

Supplementary Figure.3

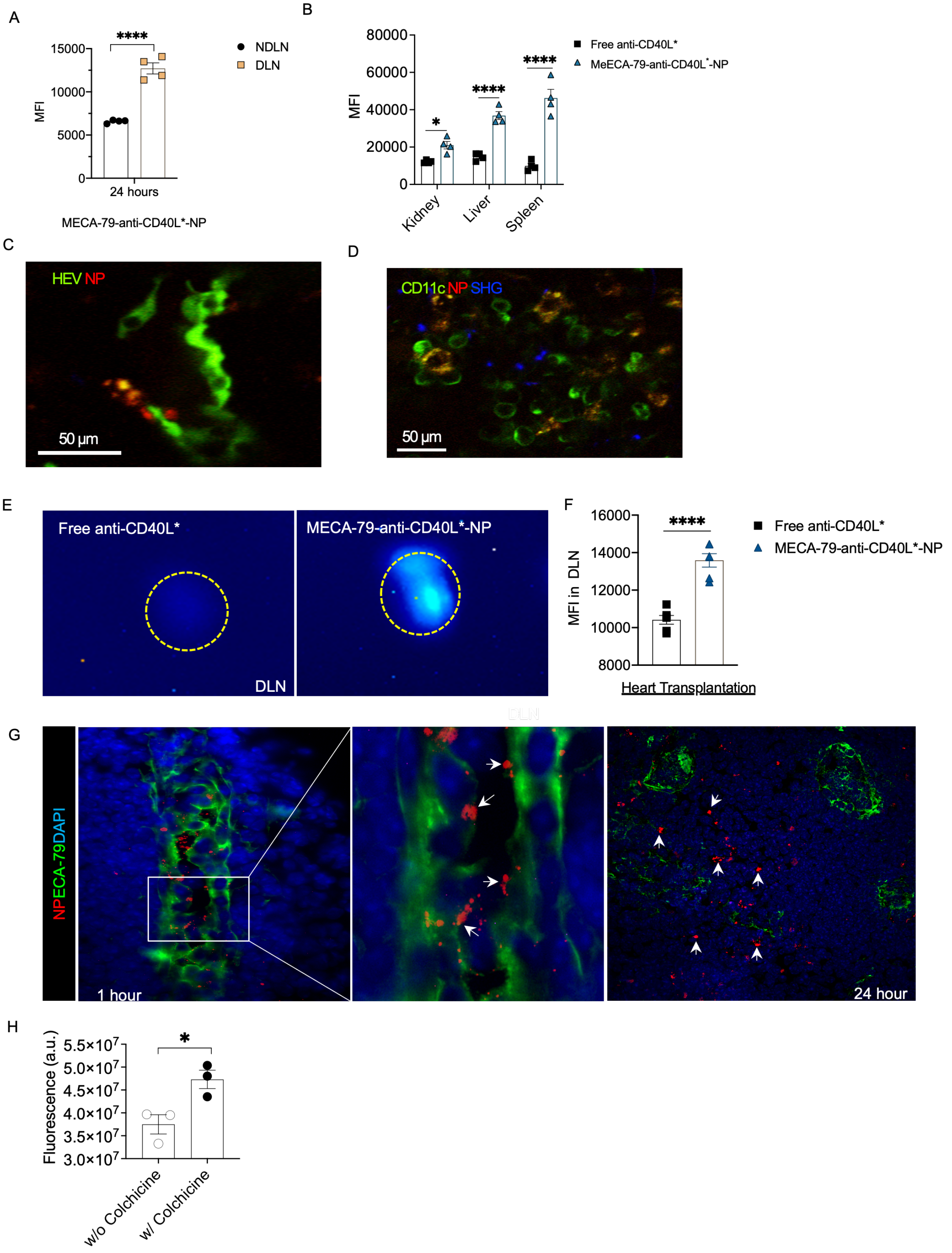

24

Supplementary Figure 3. *Characterization of anti-CD40L-NP and nanodelivery of anti-CD40L to DLNs*. (**A**) Comparison of MFI of MECA-79-anti-CD40L*-NP in DLNs and NDLNs. (**B**) Comparison between MFIs from bioluminescence of kidney, liver and spleen of free anti-CD40L*- and MECA-79-anti-CD40L*-NP-injected groups. (**C**)Intravital imaging showing MECA-79-NP (Red) and HEVs (Green) in DLN. (**D**) Intravital imaging showing MECA-79-NP (Red) and CD11c$^+$ DCs (Green) in DLN. (**E**) Live fluorescence imaging of DLNs 24 hours after administration (i.v.) of either free anti-CD40L* or MECA-79-anti-CD40L*-NP at day 8 post-transplantation. (**F**) Comparison of MFI by bioluminescence between DLNs of free anti-CD40L*- and MECA-79-anti-CD40L*-NP injected groups (n=3 mice/group). (**G**) IF staining of DLNs from mice 1 hour and 24 hours following injection of MECA-79-NP-Alexa594 (n=3 mice) (white arrows indicate NPs). (**H**) Fluorescence signal of MECA79-NP in PNAd$^+$ CHO cells after exocytosis in presence or absence of colchicine treatment (n=3/group). Data presented as mean ± SEM, *p<0.05, *** p<0.001, ****p<0.0001.

Supplementary Figure.4

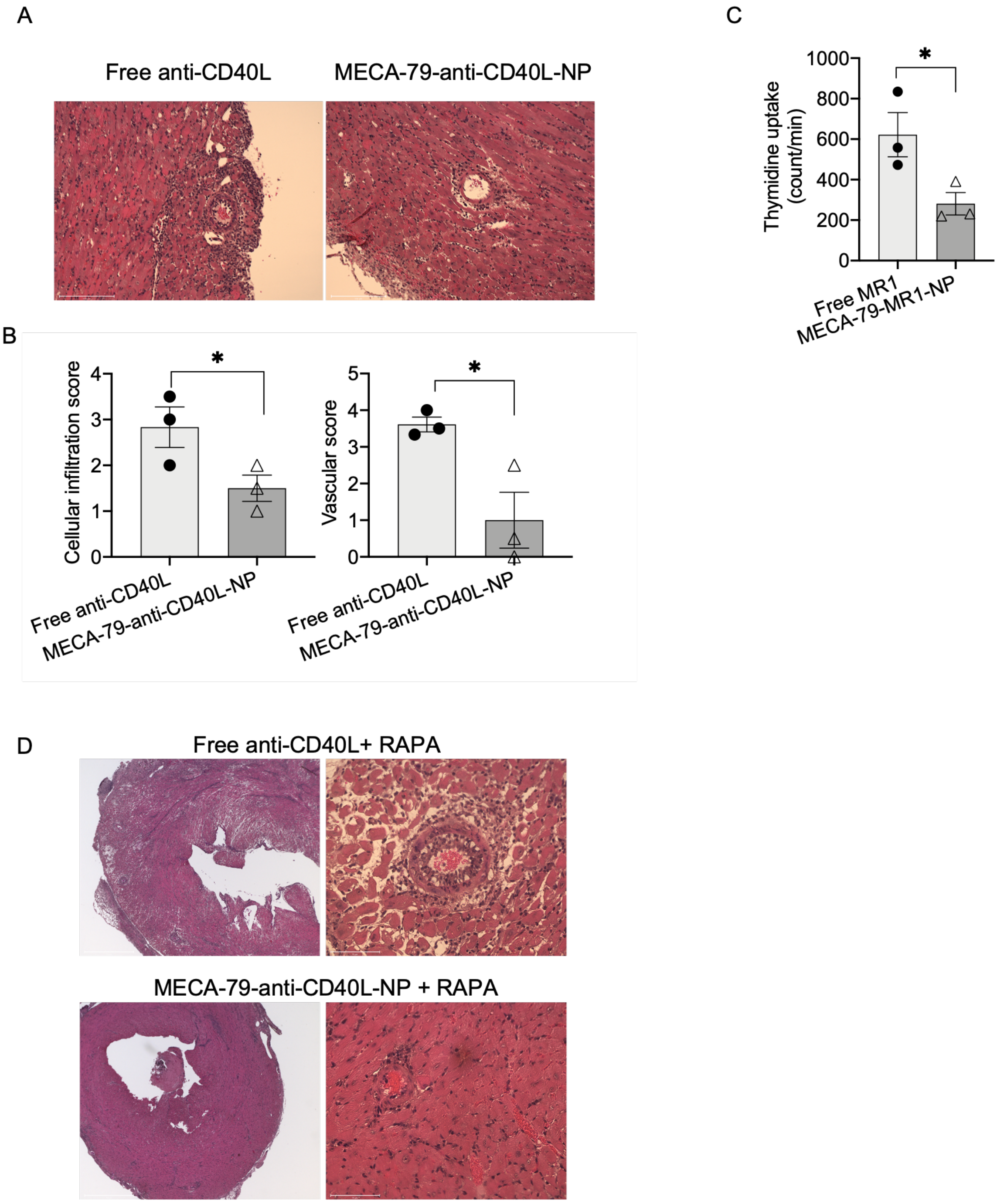

Supplementary Figure 3. *Meca-79-anti-CD40L-NP alone or in combination with rapamycin induces long-term heart allograft survival in mice.* (**A**) Representative light micrographs of H&E-stained heart allograft sections at Day 7 from WT recipients treated with free anti-CD40L or

MECA-79-anti-CD40L-NP (Scale bar: 150µm). (**B**) Comparison of cellular infiltration and vascular damage of the heart allografts in WT recipients following treatment with free anti-CD40L or MECA-79-anti-CD40L-NP (n=3 mice/group). (**C**) Comparison of T cell proliferation between free anti-CD40L or MECA-79-anti-CD40L-NP by MLR assay. (**D**) Representative light micrographs of H&E-stained heart allograft sections at day 21 post-transplantation from WT recipients treated with a combination of free anti-CD40L and RAPA or a combination of MECA-79-anti-CD40L-NP and RAPA (Scale bar:750µm, 75µm). Data presented as mean ± SEM, * $p<0.05$.
